# The impact of “flattening the curve” on Generation Z

**DOI:** 10.1016/j.ebiom.2020.103013

**Published:** 2020-09-18

Since 1966, September 8 has been declared International Literacy Day by UNESCO to highlight the importance of literacy among international communities. The United Nation's Sustainable Development Agenda, widely accepted by world leaders in September, 2015, encourages equal access to basic education and learning opportunities for all humankind. However, this notion has been widely challenged across the world because of the devastating implications of the COVID-19 pandemic this year. As many nations observe a promising trend in getting over the worst peak of the pandemic, governments are keen to kickstart the economy and continue to relax wider physical distancing measures along with consideration to re-opening of schools to allow parents who have childcare responsibilities to return to work.

In a bid to cut viral transmission during the early days of the COVID-19 pandemic, social distancing and lockdown measures included the suspension of in-person education to children and young people, apart from children of keyworkers, for some weeks to months. According to estimates by UNESCO, more than 60% of pupils around the world have experienced disruption to their education by school closures during the pandemic. There has been clear evidence of the turmoil and rising inequalities among young people when A-level and GCSE results were revealed on August 13 and August 20, respectively, in the UK. The controversial exam grading algorithm used by UK exams regulator Ofqual led to a 40% downgrading in A-level grades, resulting in nationwide outcry from pupils from state schools, whose grades were affected the most. In response, the UK Education Secretary Gavin Williamson had to take a swift U-turn to allow predicted grades to be used for exam results. This grading fiasco continued to disrupt other forms of qualification results, including BTec vocational courses, for which the negative effect is disproportionately experienced by students from BAME communities and low-income families.

Countries around the world must prioritise preparations for reopening schools to minimise the potential damage to children and young people's educational, psychological, and social development posed by further lockdown. As we gain a better understanding of the clinical symptoms and epidemiology of COVID-19, compared with influenza and other respiratory infections, children appear to be less susceptible to the severe outcome of COVID-19 infections than do older adults. If they do get infected, they typically experience mild disease. On Aug 3, 2020, in a study published in *The Lancet Child & Adolescent Health*, Kirstine Macartney and colleagues tracked SARS-CoV2 transmission among children and staff in schools and early childhood education and care settings in New South Wales, Australia, from January 25 to April 10, 2020. They identified 27 primary cases and 1448 close contacts, half of whom were tested, resulting in only 18 secondary cases. Although these low transmission rates may reflect the mitigation measures that were in place, including facility closure following case identification and home quarantine for close contacts, the results are consistent with a similar study from Ireland published in the *Journal of Euro Surveillance*, undertaken during the same period.

To add to the debate, the reopening of schools has been modelled in another study published in *The Lancet Child & Adolescent Health* on July 30. In this study, researchers fitted an individual-based model to UK-specific data and evaluated several variants to easing lockdown, including three test, trace, and quarantine approaches, and two methods to reopening schools in September. The findings indicated that reopening schools and easing of lockdown measures to pre-COVID-19 levels are likely to result in a second wave of infections unless the current testing is substantially scaled up. These studies highlight the key role that country-specific test, trace, and isolate systems will play to allow for the reopening of schools to prevent a second wave.

Despite high daily infection rates still being reported in some parts of the world, including Brazil, the USA, and India, governments around the world have started to relax lockdown measures and lift travel restrictions since the beginning of July, to stimulate their economies. However, these re-openings have, in some cases, unfortunately led to a resurgence of local community-acquired infections, as well as travel-imported cases of COVID-19, requiring renewed lockdowns, as witnessed in parts of China, Europe, Australia, New Zealand, and some US states. Testing strategies are needed to track and contain these new infections, particularly as we aim to reopen schools.

The predominant test strategy for diagnosing SARS-CoV-2 infection has involved highly accurate and sensitive RT-PCR analyses. Early in the pandemic, testing by this approach resulted in considerable bottlenecks due to the time-consuming, resource-intensive, and costly nature of the assay. More recently, modelling studies such as the one by Mirjam Kretzschmar and colleagues published in the August issue of *The Lancet Public Health* suggest that optimised tracing using app-based technologies, coupled with an effective testing approach, has the potential to prevent up to 80% of all transmission. On the basis of this and other similar studies, public health officials, scientific bodies, and epidemiologists have shifted their attention to COVID-19 tests that can offer a rapid and affordable method to repeatedly screen whole communities rather than just symptomatic individuals, alongside effective contact tracing strategies to halt transmission of the virus within local community settings.

In an Article published in our August issue, Lisa Ng and colleagues attempted to overcome the detection limit of current serological assays by identifying a novel linear B-cell immunodominant epitope-based assay to detect previous exposure to SARS-CoV-2. Two of the epitopes reached sensitivity and specificity of more than 90% for detecting SARS-CoV-2 from COVID-19-positive patients 23 days after infection; combining the two epitopes could reach 100% sensitivity and specificity, showing the potential for diagnostic-grade lateral IgG lateral flow POC test development. Antigen tests, which immobilise antibodies on a test strip for detecting viral proteins in saliva or nasal swabs offer further opportunities for scalability of repeat testing at the fraction of the cost for local surveillance. Currently, Becton Dickenson (BD) and Quidel have already obtained emergency use authorisation from the US Food and Drug Administration for their commercial use of antigen tests for SARS-CoV-2. Alternatively, government-funded approaches, such as SHERLOCK (a CRISPR-based approach that requires ligation step for the development of home test kits) and an National Institutes of Health-funded REDX project that aims to create high throughput diagnostic tests that can ramp up lab-based assay are already underway.

Aside from having rapid testing approaches for SARS-CoV-2 in place, deployment of digital public health technologies is being highlighted by governments and private sectors as strategic solutions for mitigating the COVID-19 pandemic and easing physical distancing measures. However, the ethical and legal implications of using digital tools for surveillance and control purposes are uncertain, and limitations of these tools will need careful consideration to protect personal data and intrusion of privacy, especially when public services are outsourced to private companies for pandemic management.

As the new academic year begins this Autumn, there is a need to deploy a multitude of COVID-19 test approaches. Rapid and affordable SARS-CoV-2 tests would provide opportunities for repeated testing to implement contact-tracing and isolation measures quickly, and big data-based tracing tools would help to facilitate effective seroprevalence monitoring of potentially infected cases within local communities. Both approaches require careful consideration from public health officials and policymakers. In this regard, *EBioMedicine* is proud to publish the most cutting-edge translational research tools that will accelerate the fight against the COVID-19 pandemic.

*EBioMedicine*

